# Resting network architecture of theta oscillations reflects hyper-learning of sensorimotor information in Gilles de la Tourette syndrome

**DOI:** 10.1093/braincomms/fcae092

**Published:** 2024-03-14

**Authors:** Adam Takacs, Eszter Toth-Faber, Lina Schubert, Zsanett Tárnok, Foroogh Ghorbani, Madita Trelenberg, Dezso Nemeth, Alexander Münchau, Christian Beste

**Affiliations:** Cognitive Neurophysiology, Department of Child and Adolescent Psychiatry, Faculty of Medicine, TU Dresden, Dresden 01069, Germany; Faculty of Medicine, University Neuropsychology Center, TU Dresden, Dresden 01069, Germany; Institute of Psychology, ELTE Eötvös Loránd University, Budapest 1064, Hungary; Brain, Memory and Language Research Group, Institute of Cognitive Neuroscience and Psychology, HUN-REN Research Centre for Natural Sciences, Budapest 1117, Hungary; Institute of Systems Motor Science, University of Lübeck, Lübeck 23562, Germany; Vadaskert Child and Adolescent Psychiatry Hospital and Outpatient Clinic, Budapest 1021, Hungary; Cognitive Neurophysiology, Department of Child and Adolescent Psychiatry, Faculty of Medicine, TU Dresden, Dresden 01069, Germany; Faculty of Medicine, University Neuropsychology Center, TU Dresden, Dresden 01069, Germany; Cognitive Neurophysiology, Department of Child and Adolescent Psychiatry, Faculty of Medicine, TU Dresden, Dresden 01069, Germany; INSERM, Université Claude Bernard Lyon 1, CNRS, Centre de Recherche en Neurosciences de Lyon CRNL U1028 UMR5292, Bron 69500, France; NAP Research Group, Institute of Psychology, Eötvös Loránd University & Institute of Cognitive Neuroscience and Psychology, HUN-REN Research Centre for Natural Sciences, Budapest 1071, Hungary; Department of Education and Psychology, Faculty of Social Sciences, University of Atlántico Medio, Las Palmas de Gran Canaria 35017, Spain; Institute of Systems Motor Science, University of Lübeck, Lübeck 23562, Germany; Cognitive Neurophysiology, Department of Child and Adolescent Psychiatry, Faculty of Medicine, TU Dresden, Dresden 01069, Germany; Faculty of Medicine, University Neuropsychology Center, TU Dresden, Dresden 01069, Germany

**Keywords:** connectivity, Gilles de la Tourette syndrome, statistical learning, theta, resting state

## Abstract

Gilles de la Tourette syndrome is a neurodevelopmental disorder characterized by motor and vocal tics. It is associated with enhanced processing of stimulus–response associations, including a higher propensity to learn probabilistic stimulus–response contingencies (i.e. statistical learning), the nature of which is still elusive. In this study, we investigated the hypothesis that resting-state theta network organization is a key for the understanding of superior statistical learning in these patients. We investigated the graph–theoretical network architecture of theta oscillations in adult patients with Gilles de la Tourette syndrome and healthy controls during a statistical learning task and in resting states both before and after learning. We found that patients with Gilles de la Tourette syndrome showed a higher statistical learning score than healthy controls, as well as a more optimal (small-world-like) theta network before the task. Thus, patients with Gilles de la Tourette syndrome had a superior facility to integrate and evaluate novel information as a trait-like characteristic. Additionally, the theta network architecture in Gilles de la Tourette syndrome adapted more to the statistical information during the task than in HC. We suggest that hyper-learning in patients with Gilles de la Tourette syndrome is likely a consequence of increased sensitivity to perceive and integrate sensorimotor information leveraged through theta oscillation-based resting-state dynamics. The study delineates the neural basis of a higher propensity in patients with Gilles de la Tourette syndrome to pick up statistical contingencies in their environment. Moreover, the study emphasizes pathophysiologically endowed abilities in patients with Gilles de la Tourette syndrome, which are often not taken into account in the perception of this common disorder but could play an important role in destigmatization.

## Teaser

Takacs *et al*. report that adults with Gilles de la Tourette syndrome (GTS) show better learning of probabilistic relations alongside a more optimal theta network prior to the task, which suggests that adults with GTS had an increased sensitivity to perceive and integrate novel information as a trait-like characteristic.

## Graphical Abstract

**Graphical Abstract:**
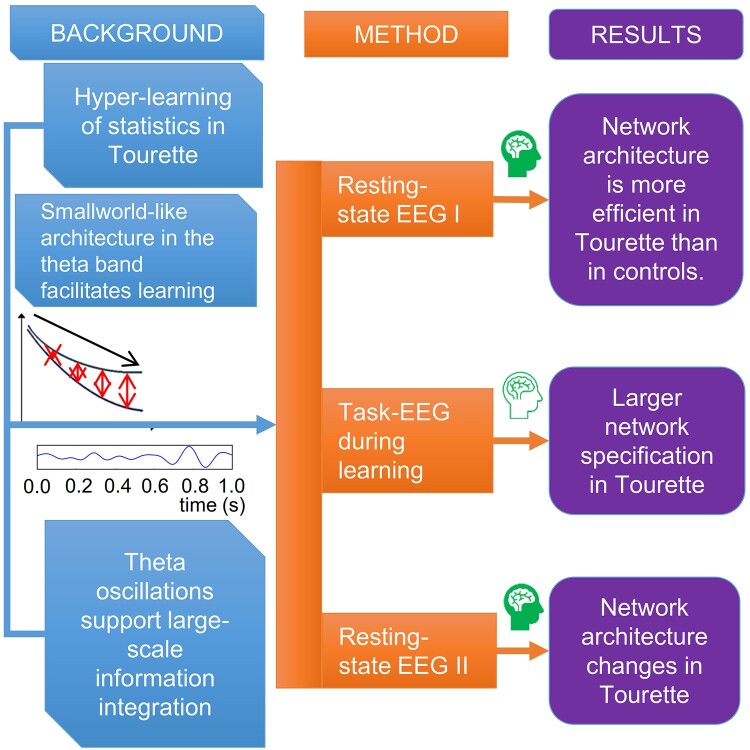

## Introduction

Motor and vocal tics are the main characteristics of Gilles de la Tourette syndrome (GTS),^1-3^ which also affects various cognitive domains, such as learning, cognitive control, social cognition and communication.^4-8^ Therefore, symptoms of GTS should be understood in the wider context of an altered information processing systems. From this perspective, tics, often seen as pure motor symptoms in GTS, probably resemble habitual actions as an expression of a higher propensity to form associations between stimuli and responses (S–R),^5,9-12^ which in turn is reflected by enhanced learning of probabilistic S–R information in these patients.^8,13,14^ The ability to extract probabilistic properties from the environment across time and space is referred to as ‘statistical learning’,^15-18^ a fundamental ability that supports acquiring and using a wide range of skills and habits. Yet, despite its clinical relevance and likely being a key to a better understanding of GTS, it is still elusive what this higher propensity of statistical learning constitutes at the neurophysiological level. We aimed to examine the role of this enhanced function in the pathophysiology of GTS by using a network perspective.

We propose that resting-state network connectivity patterns in specific oscillatory frequency bands are keys to the understanding of altered information processing in GTS: resting-state activity forms the basis for all task-related activity. During a task, resting-state activity is transformed into another brain state that maps specific requirements during a task. Several lines of evidence stress the state-dependency of neurophysiological processes underlying task performance.^19^ In particular, statistical learning processes and the rapid encoding of information necessary for learning are related to resting brain states.^20-22^ Also, changes between pre- and post-learning resting-state connectivity were suggested to reflect an early stage of memory consolidation.^23^ Therefore, it appears likely that an in-depth analysis of the organization of resting-state activity is central to better understanding ‘hyper-learning’ of S–R associations as one important feature in GTS. Notably, recent conceptual views on GTS proposed that the integration of perception and action may be key to better framing the various characteristics of GTS phenomenology,^9,11,24,25^ and it has been argued that increased (statistical) learning in GTS is connected to principles also governing enhanced perception-action integration in GTS.^12^ Of note, the integration of perception and action critically depends on the specific network organization of theta band activity.^26,27^ In addition, modulations of so-called small-world network characteristics^28-31^ well reflect perception-action integration processes,^26,27^ which have also been hypothesized to frame statistical learning.^12^

**Figure 1:**
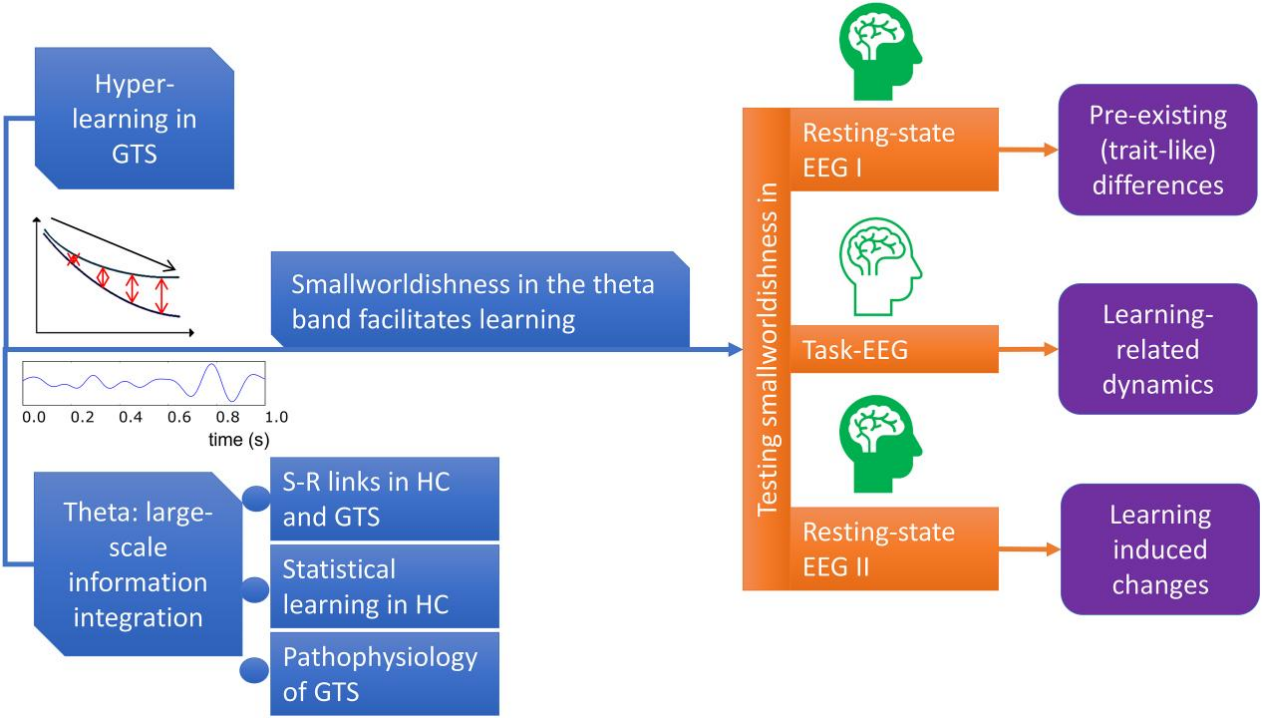
**A schematic illustration of the study rationale.** The blue boxes on the left side represent previous research that informed the current hypotheses. These include (i) behavioural evidence of enhanced learning of probabilistic stimulus–response information in GTS^8,13,14^, (ii) a biophysical perspective of why theta band activity should be considered in learning^19,35^, (iii) a clinical perspective of why theta band activity should be relevant in GTS^36^ and (iv) specific results on theta activity’s role in statistical learning^21,37,38^ and stimulus–response binding.^39,40^ Importantly, it was assumed that superior learning performance is associated with a more optimal network architecture,^32,33^ which resulted in the use of the small-world coefficient. Network architecture was assessed before, during and after the learning task (see the orange rectangles in the middle). Thus, we could conclude whether group differences in the small-world coefficient reflect pre-existing differences, only revealed during the learning period and if any difference remained observable after that (purple boxes on the right).

From a graph–theoretical perspective, the pattern of connectivity determines how efficient information processing is in a given network architecture.^28-31^ For instance, a network architecture consisting predominantly of locally connected nodes with only a few long-distance connections is considered well suited for modularized tasks. On the other hand, a deeply interwoven pattern of the connectome, including long-distance pathways, promotes information integration across the whole brain. Small-world networks keep a balance between local and global connectivity, i.e. although there are local clusters, it is still easy to reach distant nodes in the network through a small number of intermediate steps facilitating quick dissemination of information and efficient processing.^29,30^ Small-world neural networks perform faster and more accurate in learning tasks.^32^ It was suggested that this type of connectivity pattern scaffolds the network’s capabilities of learning and retention^33^: The former requires integration across distributed elements of the network, while the latter relies on segregation (i.e. specialization) within clusters of nodes.^33,34^ Against this background, the small-world network architecture in the resting state may be central to better understand processes of increased (statistical) learning in GTS. Resting-state EEG before an incidental statistical learning task can provide insight into pre-existing differences in network architecture between GTS and HC participants, i.e. how optimized their neurophysiological connectomes are to process subsequent novel information. Moreover, modulations in the small-worldness of a neurophysiological network during and after learning can be used to delineate dynamic changes in the network state (during the task) and medium-term changes that occurred after learning (second resting state). This comprehensive assessment will yield information on how capable the GTS neurophysiological connectome is to encoding novel information and how the integration of the new content shapes the network. The study’s rationale is depicted in Fig. 1.

We hypothesize that adult patients with GTS have better statistical learning than the healthy control (HC) group that is based on a more optimal network architecture in patients with GTS compared to HC, i.e. the resting-state connectome is more small-world-like in the former.^32,33^ Moreover, we assumed that statistical learning during the task changes the network architecture both during and after the task more consistently in the GTS than in the HC group. Regarding network dynamics, we focused on theta band activity for various reasons: First, high-amplitude, low-frequency oscillations, such as those in the theta band, are particularly suitable for large-scale information integration,^19,35^ including establishing perception–action associations in HC^39^ and GTS.^40^ Second, connectivity changes in the theta band have been linked to statistical learning.^37^ Third, theta band activity plays a crucial role in the pathophysiology of fronto-striatal networks in GTS,^36^ also known to underlie statistical learning.^21,38^

## Materials and methods

### Participants

A total of *n* = 26 participants with GTS and *n* = 36 neurotypical participants were recruited for our study (Table 1). One patient with GTS had to be excluded because he consistently showed low baseline accuracy (<70%) in the learning task, indicating low task engagement. Hence, 25 participants with GTS were included in the analyses (19 men, 6 women, *M*_age_ = 34.12 years, SD_age_ = 10.31 years, range between 20 and 55 years). We matched HC participants on a one-to-one basis to each GTS participant; hence, 25 HC individuals were included in our analyses (14 men, 11 women, *M*_age_ = 32.88 years, SD_age_ = 11.81 years, range between 18 and 58 years). Each participant underwent a thorough clinical assessment, including a semi-structured interview measuring tic severity (Yale Global Tic Severity Scale)^41^ and obsessive-compulsive symptoms (Yale-Brown Obsessive Compulsive Scale).^42^ In the GTS group, 10 participants had comorbid diagnoses and 13 participants were taking medication (detailed patient characteristics can be found in Table 1). None of the participants in the matched HC group had any neurological, psychiatric or neurodevelopmental disorders or took centrally acting medication. All participants had normal or corrected-to-normal vision and hearing. In both groups, the mean IQ was in the normal range and did not differ significantly between GTS and HC participants (GTS: 107.9 ± 10.11, HC: 110.0 ± 9.2, *t*(48) = −0.754, *P* = 0.455).

**Table 1:** Clinical characteristics of the patients with GTS involved in the study

Patient	Age	Sex	Disease duration (years)	DCI	YGTSS total	YGTSS tics	Rush score	Comorbidities	Medication
1	45	1	38	86	69	39	12	Depressive episode	Pimozide, biperiden
2	21	1	3	83	16	16	8		
3	25	2	21	86	33	13	14		
4	47	1	32	66	15	15	13		
5	33	1	26	64	35	25	10		
6	25	2	21	54	6	6	5		
7	48	2	43	51	31	11	8	Depression, burnout	Escitalopram, candesartan
8	28	2	23	55	31	21	16		
9	47	1	37	100	46	26	16	Depression	N/A
10	29	2	26	66	55	25	18	Borderline personality disorder, anxiety disorder, depressive episode	Alprazolam (if needed, every 2–3 weeks)
11	22	1	17	62	34	24	15		Aripiprazole
12	20	1	11	90	39	29	19		N/A
13	25	1	19	51	40	20	6	Depression	Pimozide
14	23	1	17	71	40	30	9	Depression, OCD, social phobia	Aripiprazole
15	54	1	48	65	51	21	15	Depression	Pantoprazol, paroxetine, simvastatin, korodin (if needed), mirtazapine (if needed), mometasonfuroat, sumatriptan
16	50	1	ca. 40	38	0	0	3		Aripiprazole
17	40	1	34	85	26	16	14	OCD	Citalopram
18	21	2	14	93	37	17	15		
19	37	1	32	100	57	37	14	ADHD, depressive episode	N/A
20	35	1	29	53	41	21	16	ADHD	Methylphenidate
21	30	1	24	48	18	18			
22	35	1	26	49	27	47			
23	35	1	25	45	20	20			
24	33	1	23	88	18	38			
25	42	1	17	76	25	45			
Mean	34		25.25	69	32.40	23.20	12.30		

ADHD, attention deficit hyperactivity disorder; DCI, diagnostic confidence index; OCD, obsessive-compulsive disorder; Rush, modified rush videotape rating scale; YGTSS, yale global tic severity scale.

HC participants were recruited from the volunteer pool of the Technische Universität Dresden, the University Clinic Carl Gustav Carus, the University of Lübeck in Germany and the Eötvös Loránd University in Hungary; GTS participants were recruited from the University of Lübeck in Germany and the Hungarian Tourette Syndrome Society in Hungary. The assessment procedure and EEG protocol were identical at the three measuring sites. Written informed consent was obtained from all participants before study entry. The study was performed in accordance with the declaration of Helsinki and was approved by the ethics committee of the universities.

#### Task: Alternating Serial Reaction Time (ASRT) task

**Figure 2:**
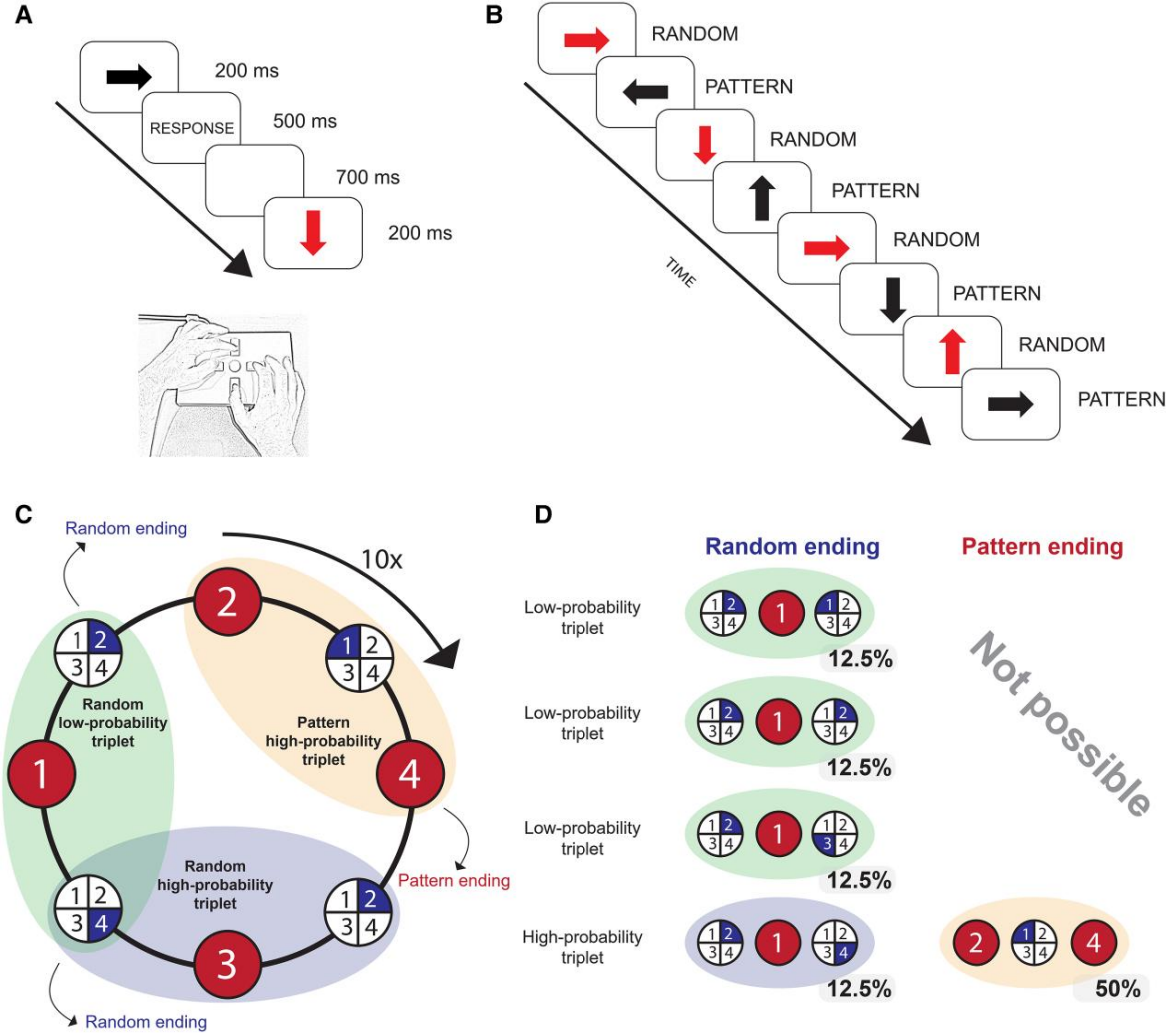
**The ASRT task.** (**A**) Arrows were presented in the middle of the screen and participants were instructed to press the corresponding response key on the response box. (**B**) The presentation of the stimuli followed an eight-element sequence in which pattern and random trials alternated with each other. (**C**) Due to the alternating sequence, pattern high-probability, random high-probability and random low-probability triplets (runs of three consecutive elements) could be differentiated. Numbers indicate the direction of the arrows (1 = left, 2 = up, 3 = down, 4 = right). Numbers in large circles with red shading represent pattern trials and numbers in partial circles with blue shading represent random trials. (**D**) Some triplets were more probable than others. The last trial of a high-probability triplet could be either pattern or random, while low-probability triplets always end with a random element. The figure was adapted from Vékony *et al*.^47^

Statistical learning was measured by the cued version of the Alternating Serial Reaction Time (ASRT) task,^43,44^ which was modified to fit the EEG measurements^45^ and shown to have high reliability to measure learning.^46^ In this version of the task, an arrow stimulus appears at the centre of the screen. Participants are asked to press the corresponding key to the spatial direction (up, down, left or right) of the arrow as accurately and as fast as they could on the Cedrus RB-540 response pad (Fig. 2A).

The presentation of the stimuli followed an eight-element alternating sequence, within which pattern and random elements alternated with each other [e.g. 1-*r*-2-*r*-3-*r*-4-*r*, where numbers refer to the four possible spatial directions (1 = left, 2 = up, 3 = down, 4 = right) of the arrow stimuli and ‘*r*’ indicates a randomly selected direction out of the four possible ones]. In the cued ASRT task, pattern and random elements are denoted by different visual stimuli: pattern elements are shown in black, whereas random elements appear in red (Fig. 2B). Participants were informed that the black arrows follow a pre-defined pattern, while the red arrows point in a random direction. Importantly, participants are only informed about the presence of the sequence, but they have no information about the exact sequence. They were asked to find the pattern of the black arrows’ spatial directions to improve their performance.

In the ASRT task, three successive elements constitute a triplet. Due to the alternating sequence presented in the task, some triplets are more likely to occur than others. In the example sequence, 1-*r*-2-*r*-3-*r*-4-*r*, 1-X-2, 2-*X*-3, 3-*X*-4 and 4-*X*-1 (where *X* refers to the middle element of the triplet) occur with a higher probability as their third elements can be either pattern or random. In contrast, 1-*X*-3 and 2-*X*-4 appear with a lower probability as their third elements can only be random. The former type of triplet is labelled as a high-probability triplet and the latter one is referred to as a low-probability triplet. Beyond the distinction of high- and low-probability triplets, another aspect of the task is the structure of the elements, i.e. whether they are pattern or random elements. High-probability triplets can be distinguished based on whether their last elements are pattern or random elements. The last element of a low-probability triplet can only be random since pattern elements can only occur with high probability (Fig. 2C).

Based on probability and structure, three trial types can be differentiated: (i) trials that are the last element of a high-probability triplet and belong to the pre-defined sequence, referred to as high-probability pattern trials, (ii) random elements that belong to high-probability triplets labelled as high-probability random trials and (iii) random elements that are the last elements of a low-probability triplet called low-probability random trials. Considering these trial types, we can define statistical learning as the difference in accuracy or reaction time (RT) between high-probability random and low-probability random trials (Fig. 2D). The sequence properties of these trials are identical as they both appear randomly, but their statistical properties are different as the former are more probable than the latter. Hence, statistical learning captures the acquisition of probability-based regularities.

The stimuli were presented in blocks, each block contained 85 trials. Each block started with five random trials for practice, then the eight-element alternating sequence was presented 10 times. The presentation of the trials was fixed-paced (Fig. 2A). The stimulus was presented at the centre of the screen for 200 ms. Then, a blank screen was shown until the participant pressed a button but no longer than 500 ms. Next, a 700-ms-long fixed delay with a blank screen was followed by the next trial. If participants gave an incorrect response, a blank screen was presented followed by an ‘*X*’ on the screen, which was presented for 500 ms at the centre of the screen. If no response was given in the 500-ms-long response window, a ‘!’ was presented at the centre of the screen for 500 ms.

#### Behavioural data analysis

Accuracy scores and reaction times were calculated for high-probability random and low-probability random trials. Trials without responses were excluded from all analyses and reaction times were calculated only for the correct responses. Trills (e.g. 1-2-1) and repetitions (e.g. 2-2-2) were also removed as participants often show pre-existing tendencies for them.^48^ The first seven trials at the beginning of each block, i.e. the first five random practice trials and the first two elements of the first triplet, were also excluded from the analysis. The remaining trials were categorized in a moving window manner throughout the stimulus stream. A given trial was first categorized as the third trial of a triplet (as a predicted item); it was then also categorized as the middle (interim item) and lastly as the first trial (as a predictor item) of the following two triplets (Fig. 2C). Crucially, performance is operationalized on the level of trials and not triplets, i.e. accuracy scores and RTs are always calculated only on the last trial of a triplet.

The stimuli were presented in blocks, each consisting of 85 trials. During the behavioural analysis, the task was analysed in larger, five-block units.^44,47^ For each participant and each five-block unit, the mean accuracy and median RT were calculated separately for high-probability random and low-probability random trials. Statistical learning is defined by the difference between responses to high-probability random and low-probability random trials. A greater difference between the respective trials indicates better learning.

#### EEG recording and pre-processing

To record the EEG during the resting states as well as during the task, 60 Ag/AgCl electrodes (EasyCap, Germany) were used with a BrainAmp amplifier (Brain Products GmbH, Gilching, Germany). The Brain Vision Recorder 1.2 software was used for data collection. The layout of the electrodes was based on the standard 10% system with equidistant scalp positions. The ground electrode’s coordinates were at *θ* = 58, *ϕ* = 78 and the reference electrode’s coordinates were at *θ* = 90, *ϕ* = 90. Impedances were kept below 10 kΩ and data was recorded at a sampling rate of 500 Hz. The recorded EEG data were pre-processed by using Automagic^49^ and EEGLAB^50^ on Matlab 2019a (The MathWorks Corp., Natick, MA, USA). First, flat channels were removed, and the recordings were re-referenced to an average reference. Second, the PREP pre-processing pipeline^51^ was applied. PREP removes line noise at 50 Hz using a multi-taper algorithm and applies a robust average reference after removing contaminations by bad channels. Flat-line, noisy and outlier channels were detected and removed. A high-pass filter of 0.5 Hz and a low-pass filter of 40 Hz were used (sinc FIR filter, order: 86).^52^ Electrooculography (EOG) artefacts were removed by following a subtraction method.^53^ Muscular and remaining eye artefacts were automatically classified and removed by using an independent component analysis–based Multiple Artefact Rejection Algorithm.^54,55^ Components containing cardiac artefacts were identified and removed by using ICLabel.^56^ Finally, all removed channels were interpolated in a spherical fashion.

#### Network analysis

Network analyses were performed using the FieldTrip toolbox^57^ and customized functions and were based on the analysis presented in Zink *et al*.^31^ The pre-processed resting-state EEG data were segmented into 2000-ms-long segments, each participant had 60 segments. The pre-processed task EEG data were segmented based on the stimulus presentation, i.e. segments were created separately for high-probability random and low-probability random trials throughout the task. Additionally, to measure the dynamics of learning, we created segments according to the time-on-task: stimulus-locked segments were created in the first half of the task, containing the first 10 blocks, and in the second half of the task, containing the last 10 blocks. This approach was based on the study of Tóth *et al*.,^37^ in which units of 10 blocks provided reliable connectivity measures in the ASRT paradigm. Note that these units are larger than the units in the behavioural analysis. Segments started at −500 ms and ended at 1000 ms relative to stimulus onset. Only segments with correct responses were included. Considering segments locked to high-probability random trials, participants had 42.84 (SD = 7.03) segments in the first half of the task and 49.67 (SD = 9.78) in the second half of the task. As for segments locked to low-probability random trials, participants had 93.76 (SD = 17.94) in the first half and 97.48 (SD = 16.72) in the second half of the task.

Network analysis was run separately for the resting-state and task EEG data. In each segment, the power spectrum was analysed in a frequency range of interest (i.e. theta at 4–7 Hz, low alpha at 8–10 Hz, high alpha at 10–13 Hz and beta at 13–30 Hz). In each frequency band, the imaginary part of the coherence spectrum was calculated for all pairs of electrodes.^58^ Then, the binary adjacency network matrices were calculated. For each pair of electrodes, coherence was defined as ‘strong’ or ‘weak’. In the former case, an unweighted and undirected connection was defined, and it was represented by 1. ‘Weak’ coherence was represented by 0. Coherence was characterized as ‘strong’ if the coherence values were above the 85th percentile, and other values were defined as representing no connections. As inter-individual differences influence the distribution of the coherence values across segments and participants, this step was carried out individually for each subject’s segment.

All electrodes represent a node in the network and the imaginary part of the coherence defines the connections, i.e. edges between the nodes. To analyse network properties, a small-world metric was employed.^59-61^ For each subject, the average number of edges from one node to all other nodes (degree, *2k*), the average shortest path length (*L*_real_) and the average clustering coefficient (*C*_real_) were calculated. The average shortest path length is quantified as the mean minimum number of edges to connect one node to any other. Concerning the clustering coefficient, a local and global one can be differentiated. The local clustering coefficient represents how close a node and its neighbours are to being a clique and is quantified as the proportion of links between the nodes within the neighbourhood divided by the number of edges that could exist between them. The global clustering coefficient was calculated as the average of the local clustering coefficients of all nodes.

Small-world coefficients were calculated as proposed by Telesford *et al*.^60^ First, completely random and completely regular one-dimensional networks were created 2000 times with a ring lattice with *n* (number of electrodes) nodes of mean degree *2k* (similar to the real network). Then, the mean path length of a random network (*L*_rand_) and the clustering coefficient of a regular network (also called lattice network, *C*_latt_) were calculated. Watts and Strogatz^61^ proposed that a network has small-world network properties if it shows a balance between regular and random networks, i.e. if the clustering coefficient is high and the average path length is short. A high clustering coefficient is characteristic of regular networks. In this case, the network is locally segregated: there is a clustered interconnectivity within groups of nodes sharing many nearest neighbours. A short mean path length is typical for random networks and it indicates high global integration: geodetic distance is short between any two nodes of the network, resulting in a shorter distance between the clusters and leading to faster information integration globally.

Therefore, small-worldness can be defined as the balance of local segregation (high clustering coefficients) and global integration (short path lengths) in neural networks.^29^ The small-world coefficient (*ω*) formula is as follows:


$$\omega =\frac{L_{\mathrm{rand}}}{L}\;-\frac{C}{C_{\mathrm{latt}}}$$

Small-world coefficients can be between −1 and 1, with negative values representing regular network properties, positive values representing random network properties, and values close to zero representing small-world networks. A larger distance from zero in any direction indicates a less optimal network organization.^29^ Importantly, the small-world coefficient depends on the density of the network: a larger number of connections typically increases clustering and reduces path length.^34^ Density is directly influenced by the number of available nodes (i.e. the 60 electrodes) and the applied threshold of strong coherence (above the 85th percentile). Thus, small-world coefficients can only be interpreted as a relative difference between groups and/or conditions. Small-world coefficients were calculated separately for each participant and each frequency band.

## Results

### Behavioural data: statistical learning

**Figure 3:**
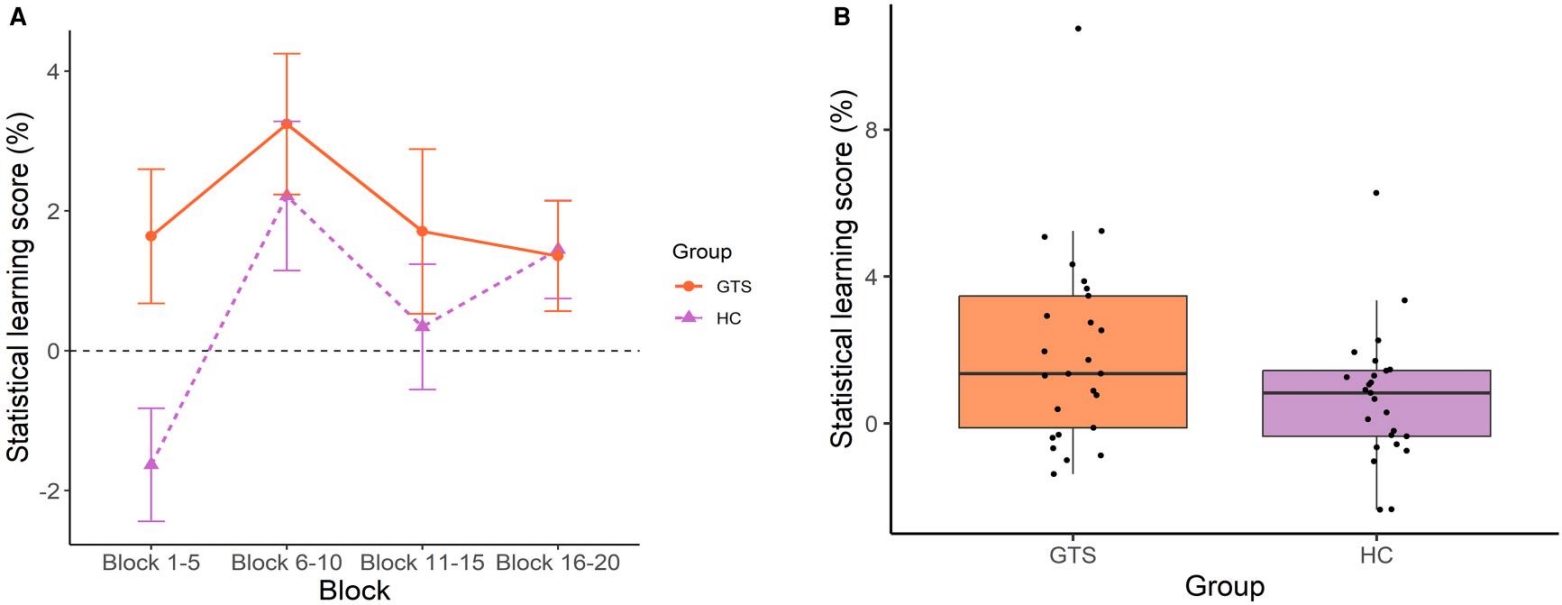
**Performance in the ASRT task.** (**A**) Statistical learning performance during the learning phase of the task. Accuracy learning scores were calculated by subtracting mean accuracy for low-probability random trials from mean accuracy for high-probability random trials in each time unit (i.e. five-block-long units) of the task. The continuous orange line with the circle symbol represents the GTS group (*n* = 25), and the dashed purple line with the triangle symbol represents the HC group (*n* = 25). The error bars denote the standard error of the mean. (**B**) Overall statistical learning performance averaged over the whole task in the GTS (with orange shading, *n* = 25) and HC (with purple shading, *n* = 25) groups. Horizontal lines within each box represent the median values, boxes extend from the 25th to the 75th percentile of each group’s distribution of values, notches show a 95% confidence interval around the median and the black circles indicate individual data points. Both panels depict the statistical learning difference between the GTS and HC groups as indicated by the significant Probability × Group interaction [*F*(1,48) = 4.032, *P* = 0.05, *η^2^_p_* = 0.077, for further details, see main text].

Statistical learning was tested with a mixed-design ANOVA on accuracy scores with Group (GTS versus HC) as a between-subjects factor and Probability (high-probability random versus low-probability random) and Block (Blocks 1–5, Blocks 6–10, Blocks 11–15 and Blocks 16–20) as within-subjects factors. Here, we report the main effect of Group and interactions involving the Group factor as they indicate differences between the GTS and HC groups. Other main effects and interactions can be found in Supplementary Table 1. The ANOVA revealed no differences in baseline accuracy scores (i.e. accuracy scores irrespective of trial types) between the GTS and HC groups [non-significant main effect of Group, *F*(1,48) = 1.474, *P* = 0.231, *η^2^_p_* = 0.030]. Importantly, the GTS and HC groups differed in the magnitude of learning (as indicated by the significant Probability × Group interaction, see details in Fig. 3). The follow-up ANOVA on the learning scores revealed that the GTS group showed higher statistical learning (*M* = 1.985%) than the HC group (*M* = 0.699%). The trajectory of statistical learning did not differ between the groups [non-significant Probability × Block × Group interaction, *F*(3,144) = 1.346 *P* = 0.262, *η^2^_p_* = 0.027]. Within the GTS group, the learning score did not correlate with tic severity [*r_s_* (23) = −0.332, *P* = 0.105].

An identical mixed-design ANOVA was run on RTs as well. The ANOVA showed no differences in baseline RTs (i.e. RTs irrespective of trial types) between the GTS and HC groups [non-significant main effect of Group, *F*(1,48) = 2.518, *P* = 0.119, *η^2^_p_* = 0.050]. We did not find any differences either in overall learning [non-significant Probability × Group interaction, *F*(1,48) = 0.718, *P* = 0.401, *η^2^_p_* = 0.015] or in the trajectory of learning [non-significant Probability × Block × Group interaction: *F*(3,144) = 1.400, *P* = 0.245, *η^2^_p_* = 0.028] between the groups. Other main effects and interactions can be found in Supplementary Table 1. Within the GTS group, the learning score did not correlate with tic severity [*r*(23) = 0.173, *P* = 0.408]. To test the possible confounding effect of medication, we ran these analyses after the exclusion of participants who took medication. Twelve GTS participants and their healthy counterparts were excluded from the analyses. The results were identical to the ones on the whole sample.

### Neurophysiological data

#### Network analyses on resting-state EEG data

The dynamics of the small-world properties were investigated on theta, low alpha, high alpha and beta oscillation-based networks separately. Here, we report the results on theta oscillation, the results on low alpha, high alpha and beta can be found in Supplementary Table 2.

**Figure 4:**
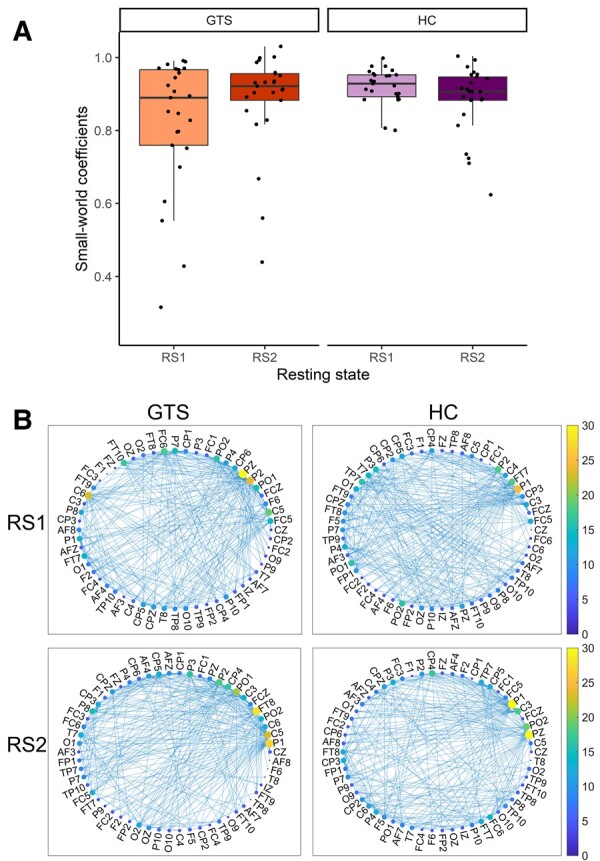
**(A) Small-world coefficients during the resting states.** The orange box plots represent the GTS group (*n* = 25), and the purple box plots represent the HC group (*n* = 25). Lighter shading indicates pre-task resting-state data (RS1), and darker shading indicates post-task resting-state data (RS2). Horizontal lines within each box represent the median values, boxes extend from the 25th to the 75th percentile of each group’s distribution of values, notches show a 95% confidence interval around the median and the black circles indicate individual data points. (**B**) Theta oscillation-based network plots during the resting states. The colour of the nodes denotes the degree of each node (i.e. how many connections this node has to any other nodes). The label of the nodes corresponds to the EEG electrode they are based on. The plots illustrate the connections (edges) with a coherence value above the 85th percentile (‘strong coherence’). Note the increase in nodes with high degree connections from RS1 to RS2 in the GTS group. Both panels depict a difference in the small-world coefficients between the GTS and HC groups as a function of time as indicated by the significant Group × Time interaction [*F*(1,48) = 4.752, *P* = 0.034, *η^2^_p_* = 0.090, for further details, see main text].

A mixed-design ANOVA on the small-world coefficient with Group (GTS versus HC) as a between-subjects factor and Time (pre-task resting state versus post-task resting state) as a within-subjects factor was conducted to test the dynamics in the small-world properties of theta oscillation-based network on resting-state EEG data. Overall, the small-world coefficient was comparable before and after the task [non-significant main effect of Time, *F*(1,48) = 0.378, *P* = 0.542, *η^2^_p_* = 0.008], and it did not differ significantly between the groups either [*F*(1,48) = 2.718, *P* = 0.106, *η^2^_p_* = 0.054]. However, there was a difference between the groups as a function of time (significant Group × Time interaction, see details in Fig. 4). The small-world coefficient on pre-task resting-state data was higher in the HC group (*M* = 0.917) than in the GTS group (*M* = 0.823, *P* = 0.016), while it did not differ on post-task resting-state data. Moreover, within the GTS group, there was an increase in the small-world coefficient after the task (*M* = 0.884) compared to that before the task (*M* = 0.823) at the trend level (*P* = 0.054). Please note that small-world coefficients in the upper range are consistent with previous analyses that used comparable electrode configurations and threshold of coherence.^26,27^

To investigate the network integration in a detailed manner, we contrasted the average shortest path length and the clustering coefficient on the resting-state data. A mixed-design ANOVA with Group (GTS versus HC) as a between-subjects factor and Time (pre-task resting-state versus post-task resting-state) as a within-subjects factor were run on the average shortest path length and the average clustering coefficient, respectively. The ANOVA on the shortest path length confirmed the difference between the GTS and HC groups [significant main effect of Group, *F*(1, 48) = 4.200, *P* = 0.046, *η^2^_p_* = 0.060] with a shorter path length in the GTS group (*M* = 2.011) than in the HC group (*M* = 2.049). The main effect of Time and the interaction did not reach significance (all *Ps* > 0.086). The ANOVA on clustering coefficient showed a difference between the GTS and HC groups at a trend level [*F*(1,48) = 3.828, *P* = 0.056, *η^2^_p_* = 0.074], with a higher clustering coefficient in the GTS group (*M* = 0.122) compared to the HC group (*M* = 0.079). Moreover, this difference changed as a function of time [significant Group × Time interaction, *F*(1,48) = 5.057, *P* = 0.029, *η^2^_p_* = 0.095]. *Post hoc* analysis revealed that the difference was more prominent in the pre-task resting-state data (*M*_GTS_ = 0.146, *M*_HC_ = 0.061, *P* = 0.006), while there was no difference between the groups in the post-task resting-state (*M*_GTS_ = 0.099, *M*_HC_ = 0.098, *P* = 0.958).

Similar to the behavioural data analyses, we ran these analyses after the exclusion of GTS participants who took medications and their HC pairs (*n* = 12 in each group). Here, contrary to the results on the whole sample, there was no observed difference between the groups in the dynamics of the small-world properties on theta oscillation-based networks; hence, medication might have influenced the results on the whole sample.

#### Network analyses on task EEG data

Analyses on small-world properties were conducted on task EEG data as well, for stimulus-locked EEG data related to statistical learning. Small-world coefficients were examined on theta, low alpha, high alpha and beta oscillation-based networks separately. Here, we report the results on theta oscillation, the results on low alpha, high alpha and beta can be found in Supplementary Tables 2 and 3. From these analyses, three HC participants had to be excluded because they did not have enough segments in the second half of the task (i.e. Blocks 11–20) after the pre-processing of the task EEG data.

**Figure 5:**
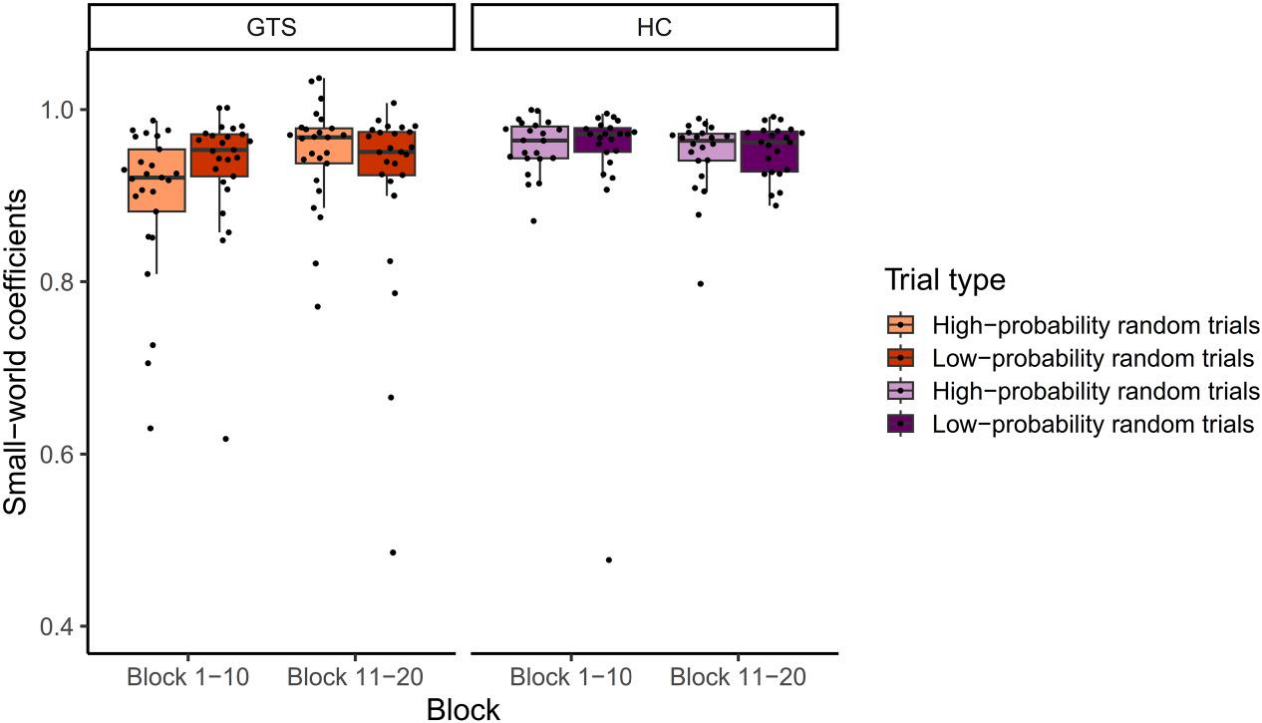
**Small-world coefficients during the task.** The orange box plots represent the GTS group (*n* = 25), and the purple box plots represent the HC group (*n* = 22). Lighter shading indicates small-world coefficients for EEG data locked to high-probability random trials and darker shading indicates small-world coefficients for EEG data locked to low-probability random trials. Horizontal lines within each box represent the median values, boxes extend from the 25th to the 75th percentile of each group’s distribution of values, notches show a 95% confidence interval around the median and the black circles indicate individual data points.

**Figure 6:**
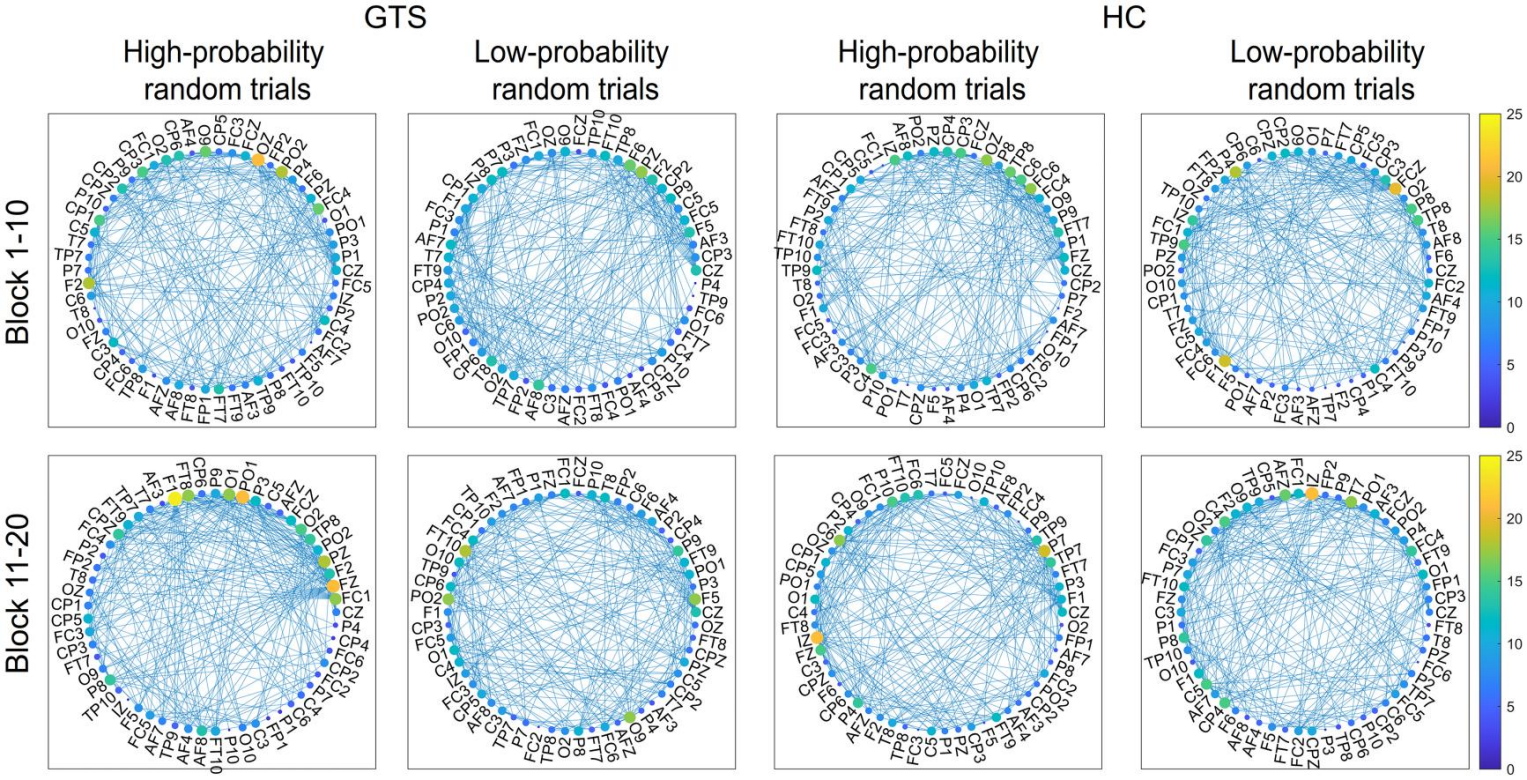
**Theta oscillation-based network plots during the task.** The colour of the nodes denotes the degree of each node (i.e. how many connections this node has to any other nodes). The label of the nodes corresponds to the EEG electrode they are based on. The plots illustrate the connections (edges) with a coherence value above the 85th percentile (‘strong coherence’). The figure depict a difference between the GTS and HC groups as a function of trial types and task blocks as indicated by the significant Probability × Block × Group interaction [*F*(1,45) = 5.210, *P* = 0.027, *η^2^_p_* = 0.104, for further details, see main text].

A mixed-design ANOVA on the small-world coefficient with Group (GTS versus HC) as a between-subjects factor and Probability (high-probability random versus low-probability random) and Block (Block 1–10 versus 11–20) as within-subjects factors was conducted to test the dynamics in the small-world properties of theta oscillation-based network on stimulus-locked EEG data related to statistical learning. We report the main effect of Group and interactions involving the Group factor in the main text as group differences are the main focus of our paper. Other main effects and interactions can be found in Supplementary Table 3. Mean network efficiency showed a difference between the two groups [significant main effect of Group, *F*(1,45) = 5.224, *P* = 0.027, *η^2^_p_* = 0.104]. *Post hoc* analysis revealed that the small-world coefficient was lower in the GTS group (*M* = 0.923) than in the HC group (*M* = 0.950), indicating a more efficient (i.e. more small-world) network in the GTS group during the task. Moreover, we found differences between the groups as a function of trial types and task blocks as well (significant Probability × Block × Group interaction, see details in Figs 5 and 6). *Post hoc* analyses revealed that in the HC group, stimulus-locked small-world coefficients did not change significantly either for high-probability random or for low-probability random trials from Blocks 1–10 to Blocks 11–20 (*P* > 0.601). In contrast, the GTS group showed lower small-world coefficients for high-probability random trials in Blocks 1–10 (*M* = 0.895) than in blocks 11–20 (*M* = 0.949, *P* = 0.005), while small-world coefficients for low-probability random trials were comparable in the two task halves (*P* = 0.423).

To investigate the network integration in a detailed manner, we also compared the average shortest path length and the clustering coefficient on the stimulus-locked data. Two ANOVAs with Group (GTS versus HC) as a between-subjects factor and Probability (high-probability random versus low-probability random) and Block (blocks 1–10 versus blocks 11–20) as within-subjects factors were conducted on the average shortest path length and the average clustering coefficient, respectively. The ANOVA on the shortest path length did not reveal any differences between the groups, trial types or blocks (all *Ps* > 0.097). The ANOVA on clustering coefficient confirmed a difference between the GTS and HC groups (significant main effect of Group, *F*(1,45) = 4.332, *P* = 0.043, *η^2^_p_* = 0.088). The clustering coefficient was higher in the GTS (*M* = 0.069) than in the HC group (*M* = 0.048), indicating higher local segregation during the task in the GTS group. Other main effects or interactions did not reach significance (all *Ps* > 0.057).

These analyses were also conducted after the exclusion of participants who took any medication. In the medication-free sample, 12 GTS participants and their HC control counterparts were included. In contrast to the original analysis, mean network efficiency differed between the two groups only at a trend-level. *Post hoc* analysis showed lower small-world coefficients in the GTS group than in the HC group, which is in line with the results of the whole sample. However, the three-way Probability × Block × Group interaction did not reach significance.

## Discussion

We investigated how the (resting) neurophysiological network architecture in GTS reflects the extraction and integration of novel sensorimotor information. While participants were unaware that the presented stimuli followed probabilistic regularities, as time progressed, differences in accuracy and RTs increased between less probable and more probable trial types. That is, statistical learning occurred incidentally in the entire sample. However, the statistical learning score was larger in the GTS than in the HC group, confirming our hypothesis of hyper-learning in GTS.^8,12-14^ Moreover, patients with GTS showed a more efficient theta band network at rest that adapted to the novel information with a gradual specialization during learning. In the following, we delineate how these results could advance our understanding of the pathophysiology of GTS.

We employed a graph theory-based approach to analyse the efficacy of information processing at the connectome level before, during and after the learning task. The efficacy of the network was operationalized as a distance from an optimal small-world coefficient. Before learning, GTS participants showed more small-world-like network architecture than HC adults in the theta band. This suggests that the GTS group was more equipped to integrate and evaluate novel information as a trait-like characteristic. Interestingly, the small-world coefficient was higher (i.e. the network architecture was more random) in the post-task resting state than before learning for GTS patients, while it did not change for HC participants. This shows that learning-induced changes in the network architecture were more persistent than transient in GTS. This was corroborated by the *post hoc* analysis showing that changes between resting states occurred through the modulation of the clustering coefficient. As the clustering coefficient represents the degree of functional segregation,^28,29^ changes in this metric suggest that the network organization in GTS was altered by specialized processing in densely connected clusters. Thus, it is possible that changes in resting networks of GTS are the consequence of superior learning during the task. This is even more relevant considering that only a few minutes break was inserted between learning and the second resting state. Thus, only rapid micro-consolidation could take place in such a short time window.^62,63^ Understanding how long-term consolidation shapes the network architecture in GTS would be of clinical importance. At the behavioural level, intact statistical knowledge was shown 1 year after the initial learning session in GTS.^64^ Thus, altered learning (and consolidation) in GTS has the potential to influence day-to-day behaviour on a longer time scale.

During the task phase, learning induced larger changes in GTS than in HC. Specifically, the small-world coefficient of more probable trials increased during the task in the GTS group, while it remained unchanged among controls. According to the follow-up analysis, the network architecture in GTS had a higher clustering coefficient than in HC throughout the task. That is, GTS participants presented higher local segregation during learning. A more segregated network favours modularized information processing rather than widescale integration.^28-31^ Taken together, GTS participants had an optimal (i.e. more small-world) theta network to encode novel information before the task was presented. Additionally, the superior efficacy to learn statistical information was also reflected by an enhanced specification (i.e. higher clustering coefficient) of the connectome during the task. Thus, statistical learning during the task changes the theta band network architecture both during and after the task more pervasively in the GTS than in the HC group. As network-level differences were already present before the task, we propose that the altered connectome in GTS is not restricted to statistical learning but reflects a special way of processing sensorimotor information. In addition, enhanced performance was shown in GTS in the binding of S–R features,^11,24^ habit formation,^5,65^ recalling words that have a strong motor association (e.g. hammer)^66^ and using language rules that were supported by statistical learning.^66,67^ The commonality of all these functions is that they involve working with representations based on sensorimotor association. In contrast, similar functions based on purely motor associations, such as response–response (R–R) binding^68^ and learning of response sequences,^69^ seem to be unchanged in GTS. The difference between enhanced processing of S–R information and intact R–R operations supports the notion that GTS is not a pure motor disorder.^9,24,68^

Crucially, we only found altered network architecture in GTS in the theta band. Analyses in other frequency bands (low alpha, high alpha, beta) did not reveal group differences in network architecture and/or learning-induced changes in the connectome (compare Supplementary material). The specificity of changes in the theta band in the current study is in line with our hypotheses and other findings that suggested theta connectivity during a task^37^ and at rest^21,38^ to be associated with encoding S–R contingencies. This is the first study to connect small-worldness in the theta band to statistical learning. Therefore, we did not only confirm that theta oscillatory activity plays a role in this cognitive domain, but also that the efficiency of the theta network architecture determines the success of learning. Interestingly, a similar role of the theta network organization has been shown before in S–R binding,^27^ another domain in which GTS participants outperform controls.^11^ The current results also fit into a broader picture in which theta synchrony across regions is considered a central mechanism of information accumulation, encoding and gating processes.^19,20,70^ From this perspective, theta oscillations are essential in connecting cortical and subcortical networks.^20^ Cortico-subcortical connectivity was implicated in both statistical learning^71^ and S–R binding processes.^19,72^ Moreover, altered connectivity between subcortical and cortical structures is considered one of the hallmarks of GTS pathophysiology.^73,74^ Within that network, theta oscillations are probably crucial in the pathophysiology of GTS.^36,75^ Thus, it is conceivable that atypical theta synchronization between distributed cortical and subcortical networks in GTS reflects an explanation for hyper-functioning in both S–R binding and statistical learning.

While the current results fit into the wider picture of enhanced functionality in GTS (including statistical and habit learning, stimulus–response binding and language), the question remains of how they contribute to understanding impairments. It is possible that when considering both enhanced and impaired cognition in GTS, we see two sides of the same coin. Namely, atypical network dynamics have been shown in GTS^76^: network hubs such as the precentral gyrus, temporo-occipital fusiform cortex and the caudate switch communication to different subnetworks over time. These shifts create an unstable network architecture, in which misinterpreted information is transferred more readily to other subnetworks. It was suggested^76^ that low network stability contributes to tics by transmitting incorrectly processed social signals to motor networks. This is in line with Albin’s social decision-making dysfunction hypothesis.^4^ At the same time, the propensity of dynamic network changes, such as decoupling between anterior and central nodes, is beneficial for statistical learning.^37^ Thus, hyper-learning in GTS might be the by-product of low network stability but not functionally relevant for tics. This explanation contrasts with the perspective that tics may reflect abnormal habit-learning mechanisms^5,77^ or enhanced stimulus–response binding.^9,11^ The current study was not meant to distinguish between the different explanations of tics. Nevertheless, we suggest that an atypical network architecture in GTS enables enhanced statistical learning. Considering statistical learning’s importance in skills and habits,^17,44^ a better understanding of how this function works differently in GTS is a relevant question irrespective of whether the social decision-making dysfunction^4^ or the stimulus-response integration^9,11^ perspectives are better models of tics.

There was no significant correlation between statistical learning and symptom severity, which suggests that hyper-learning is not directly relevant to the known clinical profile of GTS as currently examined in clinical practice and shows that the processes uncovered in the current study reflect a new facet of GTS. Notably, the heterogeneity of the GTS group might have influenced the results (e.g. medication effects on the network analyses). Nevertheless, converging evidence of hyper-learning^8,12-14^ could shed light on the often-neglected abilities conferred by the pathophysiology of GTS. Interest in and attention to GTS in social media has considerably gained momentum during the COVID-19 pandemic increasing public awareness.^78^ However, problematic misinformation including popular social media channels depicting GTS as a fringe disorder characterized predominantly by coprophenomena has considerably distorted the public image.^78^ Importantly, the fact that people with GTS not only have potentially troublesome symptoms (tics) but are also characterized by a propensity for statistical learning, which can be advantageous in certain situations and professions, is less well known.^12^ Statistical learning is a fundamental ability that enables learning of languages, music, sports and other skills.^15-18^ Understanding how children and adults with GTS could better rely on their hyper-learning in academic and everyday skills could also contribute to destigmatizing the disorder.

## Conclusion

Our study provided a novel and comprehensive analysis of the neural mechanisms of statistical learning in GTS. We found that adults with GTS exhibit enhanced statistical learning compared to HCs. It demonstrates that GTS is characterized not only by potentially troublesome symptoms (tics) but also by a propensity for hyper-learning, which can be advantageous in certain situations and professions. Superior learning performance in GTS is associated with a more efficient theta band network architecture, corroborating studies that suggested theta connectivity to be associated with encoding sensorimotor contingencies. Importantly, the theta network organization in the GTS group was already more optimal for integrating novel information in the resting state before learning than in the HC group. We propose that hyper-learning in GTS is a consequence of the altered sensitivity to process sensorimotor information.

## Supplementary Material

fcae092_Supplementary_Data

## Data Availability

The codes for processing raw behavioural data and for the network analyses as well as the reported behavioural and small-world data are available on the Open Science Framework website (https://osf.io/34ptf/?view_only=ef02205d6a044b11ab4989d845a7c8b4). EEG data in various formats (raw, pre-processed, segmented, etc.) will be provided upon request. The study has not been pre-registered.
