# Supplementary material for: Resting network architecture of theta oscillations reflects hyper-learning of sensorimotor information in Gilles de la Tourette syndrome
Source: Brain Commun. 2024 Mar 14;6(2):fcae092. doi: 10.1093/braincomms/fcae092 (PMC10984574; doi:10.1093/braincomms/fcae092)
Supplement: fcae092_Supplementary_Data [file fcae092_supplementary_data.docx]

**Supplementary Materials**

**Supplementary Table 1.** Results of the behavioural data analyses.

| Main effects and interactions | *F* | *p* | *η^2^_p_* |
| --- | --- | --- | --- |
| Accuracy data | | | |
| Block | 0.623 | 0.601 | 0.013 |
| Probability | 17.558 | < 0.001 | 0.268 |
| Group | 1.474 | 0.231 | 0.030 |
| Block × Probability | 2.886 | 0.038 | 0.057 |
| Block × Group | 0.314 | 0.815 | 0.006 |
| Probability × Group | 4.032 | 0.05 | 0.077 |
| Block × Probability × Group | 1.346 | 0.262 | 0.027 |
| RT data | | | |
| Block | 30.809 | < 0.001 | 0.391 |
| Probability | 149.934 | < 0.001 | 0.0.757 |
| Group | 2.518 | 0.119 | 0.050 |
| Block × Probability | 13.665 | < 0.001 | 0.222 |
| Block × Group | 0.972 | 0.408 | 0.020 |
| Probability × Group | 0.718 | 0.401 | 0.015 |
| Block × Probability × Group | 1.40 | 0.245 | 0.028 |

*Notes.* On accuracy data, the significant Block × Probability interaction suggests that statistical learning changed as the task progressed: post hoc analyses revealed that participants did not differentiate between high-probability random and low-probability random trials in blocks 1-5 (*M*_high-probability random_ = 92.376%, *M*_low-probability random_ = 92.373%, p = 0.996), learning became significant in blocks 6-10 (*M*_high-probability random_ = 94.373%, *M*_low-probability random_ = 91.645%, p < 0.001), did not reach significance in blocks 11-15 (*M*_high-probability random_ = 93.015%, *M*_low-probability random_ = 91.991%, p = 0.173), and was significant in blocks 16-20 (*M*_high-probability random_ = 93.303%, *M*_low-probability random_ = 91.691%, *p* = 0.004).

**Supplementary Table 2.** Results of the network analysis on resting-state EEG data.

| Main effects and interactions | *F* | *p* | *η^2^_p_* |
| --- | --- | --- | --- |
| Low alpha | | | |
| Time | 1.83 | 0.18 | 0.04 |
| Group | 0.356 | 0.554 | 0.007 |
| Group × Time | 0.899 | 0.348 | 0.018 |
| High alpha | | | |
| Time | 2.017 | 0.162 | 0.040 |
| Group | 1.080 | 0.304 | 0.022 |
| Group × Time | 0.748 | 0.391 | 0.015 |
| Beta | | | |
| Time | 1.753 | 0.192 | 0.035 |
| Group | 0.786 | 0.38 | 0.016 |
| Group × Time | < 0.001 | 0.996 | < 0.001 |

**Supplementary Table 3.** Results of the network analysis on task-EEG data related to statistical learning.

| Main effects and interactions | *F* | *p* | *η^2^_p_* |
| --- | --- | --- | --- |
| Theta | | | |
| Probability | 0.019 | 0.892 | < 0.001 |
| Block | 0.628 | 0.432 | 0.014 |
| Group | 5.224 | 0.027 | 0.104 |
| Probability × Block | 1.603 | 0.212 | 0.034 |
| Probability × Group | 0.085 | 0.772 | 0.002 |
| Block × Group | 0.604 | 0.441 | 0.013 |
| Probability × Block × Group | 5.210 | 0.027 | 0.104 |
| Low alpha | | | |
| Probability | 0.001 | 0.974 | < 0.001 |
| Block | 0.349 | 0.558 | 0.008 |
| Group | 0.276 | 0.602 | 0.006 |
| Probability × Block | 0.840 | 0.364 | 0.018 |
| Probability × Group | 1.949 | 0.170 | 0.042 |
| Block × Group | 0.285 | 0.596 | 0.006 |
| Probability × Block × Group | 0.623 | 0.434 | 0.014 |
| High alpha | | | |
| Probability | 0.021 | 0.886 | < 0.001 |
| Block | 0.969 | 0.330 | 0.021 |
| Group | 0.050 | 0.825 | 0.001 |
| Probability × Block | 0.097 | 0.757 | 0.002 |
| Probability × Group | 0.733 | 0.397 | 0.016 |
| Block × Group | 1.503 | 0.227 | 0.032 |
| Probability × Block × Group | 0.399 | 0.531 | 0.009 |
| Beta | | | |
| Probability | 0.218 | 0.643 | 0.005 |
| Block | 0.298 | 0.588 | 0.007 |
| Group | 0.598 | 0.443 | 0.013 |
| Probability × Block | 0.141 | 0.709 | 0.003 |
| Probability × Group | 0.010 | 0.920 | < 0.001 |
| Block × Group | 0.411 | 0.525 | 0.009 |
| Probability × Block × Group | 0.117 | 0.734 | 0.003 |
